# A Systematic Review of Methodological Variation in Healthcare Provider Perspective Tuberculosis Costing Papers Conducted in Low- and Middle-Income Settings, Using An Intervention-Standardised Unit Cost Typology

**DOI:** 10.1007/s40273-020-00910-w

**Published:** 2020-05-04

**Authors:** Lucy Cunnama, Gabriela B. Gomez, Mariana Siapka, Ben Herzel, Jeremy Hill, Angela Kairu, Carol Levin, Dickson Okello, Willyanne DeCormier Plosky, Inés Garcia Baena, Sedona Sweeney, Anna Vassall, Edina Sinanovic

**Affiliations:** 1 Health Economics Unit, School of Public Health and Family Medicine, Faculty of Health Sciences, University of Cape Town, Anzio Road, Cape Town, South Africa; 2 Department of Global Health and Development, Faculty of Public Health and Policy, London School of Hygiene and Tropical Medicine, London, UK; 3 Institute for Health Policy Studies, University of California, San Francisco, CA USA; 4 Department of Global Health, University of Washington, Seattle, WA USA; 5 Avenir Health, Glastonbury, CT USA; 6 TB Monitoring and Evaluation (TME), Global TB Programme, The World Health Organization, Geneva, Switzerland

## Abstract

**Background:**

There is a need for easily accessible tuberculosis unit cost data, as well as an understanding of the variability of methods used and reporting standards of that data.

**Objective:**

The aim of this systematic review was to descriptively review papers reporting tuberculosis unit costs from a healthcare provider perspective looking at methodological variation; to assess quality using a study quality rating system and machine learning to investigate the indicators of reporting quality; and to identify the data gaps to inform standardised tuberculosis unit cost collection and consistent principles for reporting going forward.

**Methods:**

We searched grey and published literature in five sources and eight databases, respectively, using search terms linked to cost, tuberculosis and tuberculosis health services including tuberculosis treatment and prevention. For inclusion, the papers needed to contain empirical unit cost estimates for tuberculosis interventions from low- and middle-income countries, with reference years between 1990 and 2018. A total of 21,691 papers were found and screened in a phased manner. Data were extracted from the eligible papers into a detailed Microsoft Excel tool, extensively cleaned and analysed with R software (R Project, Vienna, Austria) using the user interface of RStudio. A study quality rating was applied to the reviewed papers based on the inclusion or omission of a selection of variables and their relative importance. Following this, machine learning using a recursive partitioning method was utilised to construct a classification tree to assess the reporting quality.

**Results:**

This systematic review included 103 provider perspective papers with 627 unit costs (costs not presented here) for tuberculosis interventions among a total of 140 variables. The interventions covered were active, passive and intensified case finding; tuberculosis treatment; above-service costs; and tuberculosis prevention. Passive case finding is the detection of tuberculosis cases where individuals self-identify at health facilities; active case finding is detection of cases of those not in health facilities, such as through outreach; and intensified case finding is detection of cases in high-risk populations. There was heterogeneity in some of the reported methods used such cost allocation, amortisation and the use of top-down, bottom-up or mixed approaches to the costing. Uncertainty checking through sensitivity analysis was only reported on by half of the papers (54%), while purposive and convenience sampling was reported by 72% of papers. Machine learning indicated that reporting on ‘Intervention’ (in particular), ‘Urbanicity’ and ‘Site Sampling’, were the most likely indicators of quality of reporting. The largest data gap identified was for tuberculosis vaccination cost data, the Bacillus Calmette–Guérin (BCG) vaccine in particular. There is a gap in available unit costs for 12 of 30 high tuberculosis burden countries, as well as for the interventions of above-service costs, tuberculosis prevention, and active and intensified case finding.

**Conclusion:**

Variability in the methods and reporting used makes comparison difficult and makes it hard for decision makers to know which unit costs they can trust. The study quality rating system used in this review as well as the classification tree enable focus on specific reporting aspects that should improve variability and increase confidence in unit costs. Researchers should endeavour to be explicit and transparent in how they cost interventions following the principles as laid out in the Global Health Cost Consortium’s Reference Case for Estimating the Costs of Global Health Services and Interventions, which in turn will lead to repeatability, comparability and enhanced learning from others.

**Electronic supplementary material:**

The online version of this article (10.1007/s40273-020-00910-w) contains supplementary material, which is available to authorized users.

## Key Points for Decision Makers

**Table:** 

Tuberculosis (TB) unit cost data are available for low- and middle-income countries; however, there is methodological variability in how this is reported.
There are gaps in the current tuberculosis unit cost literature, particularly for 12 high TB burden countries, TB vaccinations and the interventions of above-service costs, tuberculosis prevention, and active and intensified case finding.
Going forward we need to consistently define interventions and follow standard methods to align work.

## Introduction

The epidemic of tuberculosis (TB) remains a global challenge, which has persisted despite longstanding efforts to eliminate it as a public health problem [1]. Substantial TB investment requires economic analysis for guidance and justification; however, this is often constrained by both a lack of data and a high level of resources needed to produce the necessary cost data [2]. Furthermore, easily accessible TB unit cost data are essential for many purposes such as National Strategic Planning, budgeting, priority setting and others [3]. However, variable quality of unit cost data may lead to poor decision making due to biased results. Quality can relate to robustness, precision and reliability of the data, as well as standard reporting of cost methods and results. If quality of reporting is high, comparability across cost estimates and settings is enhanced. Conversely, if reporting quality is low it is difficult to use cost estimates appropriately. In order to improve the nature and use of cost data in priority setting and decision making, understanding the quality of cost data and reporting can help to inform which methods need strengthening and which areas of reporting should be standardised.

Here we present a systematic review of papers reporting TB unit costs from a healthcare provider perspective. The working definition used in this paper for a unit cost is a cost per output (calculated through top-down, bottom-up or mixed methods), which is calculated by dividing the total cost by the quantity of output (examples of unit costs could be the ‘cost per patient treated’, the ‘cost per test’ or the ‘cost per patient retained’) [4]. Papers were assessed using study quality index indicators that included reporting standards, key cost components, precision and bias [5]. We aimed to summarise, compile and analyse all relevant historical TB cost data in low- and middle-income settings, which could be used to assess the current quality of TB cost data, highlight data gaps in research, inform necessary standards and methods, and be presented as an open access Unit Cost Study Repository on the Global Health Cost Consortium (GHCC) website [6], allowing for the addition of future TB costing work in a standardised way. The focus on low- and middle-income countries was with the intention of aiding decision makers, aiming to uncover available TB unit costs for countries where there is often thought to be a general lack of data. Specifically, the objectives were (1) to descriptively review papers reporting TB unit costs from a provider perspective in low- and middle-income settings, looking at methodological variation; (2) to assess the quality of the standards and methods used for TB unit cost papers with a study quality rating system and investigate the indicators of reporting quality through machine learning; and (3) to highlight and discuss the data gaps to inform intervention-standardised targeted TB unit cost collection going forward.

## Methods

### Search Strategy

A systematic review was conducted using disease- and intervention-specific key words relating to cost, tuberculosis and tuberculosis health services including tuberculosis treatment and prevention, which were combined using Boolean operators (see Table 1). We included papers (the term ‘papers’ is used to inclusively refer to the reports, studies, records and articles searched for and retrieved in this review) reporting TB unit costs published between January 1990 and March 2018, with no language or geographical restriction. Eight electronic databases were searched (PubMed, EMBASE, Medline, Econlit, The National Health Service Economic Evaluation Database and The Cost-Effectiveness Analysis Registry, between May and July 2016; Web of Science and Literatura Latino-Americana e do Caribe em Ciências da Saúde in February and March 2017; and through focused Google searches between November 2017 and March 2018). We excluded papers if they had no empirically collected cost data or if the currency or costing perspective could not be identified either from the paper or by contacting the authors. The search results from each database were downloaded into one library in EndNote X8 (Clarivate Analytics).

**Table 1:** Tuberculosis search terms

Category	Search terms
Cost	Cost* or economic or finance AND
Tuberculosis	TB or tuberculosis or MDR#TB or XDR#TB or multi? Drug or “resistant tuberculosis” or “strain resistance” or “mycobacterium tuberculosis” AND
Tuberculosis health services including tuberculosis treatment and prevention	treatment or management or drugs or medication or DOTS or “directly observed treatment” or “health system*” or “hospital care” or “epidemiology” or “government hospital setting” or “community based care” or “patient* perspective” or “isoniazid preventive therapy” or “IPT” or “prevention”

The asterisk (*) refers to the Boolean search modifier, where words will match if they start with the word in front of the asterisk. The hashtag (#) allows for ‘stemming’ where the beginning is similar but there may be a different suffix after the hashtag. A question mark (?) is used to match only one character

The screening of these papers was undertaken in three stages: by title, by abstract and by full text. In stage one, a researcher reviewed all papers by title (15,161), while a second researcher assessed the papers that were excluded based on title screening. Titles that identified animal research led to paper exclusion. In stage two, the resulting 6307 abstracts were screened independently by two researchers and were excluded if they did not report unit costs. In stage three, the 704 papers deemed eligible for full-text review (3% yield from the original search) were screened independently by two researchers. Papers were excluded if the studies were conducted in high-income countries based on World Bank categorisation for 2017 (*n* = 177); if no empirical data were collected regarding prices or quantities or TB-related costs (relevant data) were reported (*n* = 313), for instance if all the data were modelled or from secondary data sources; if the paper was only a correspondence, editorial, commentary, news piece or protocol (*n* = 41) or if it contained duplicate cost data (*n* = 3). We also cross-checked papers against a recent systematic literature review of TB costs for health services and patients [7].

In addition to the published literature search, we searched for grey literature, including focused Google searches, which yielded a total of 398 potential papers. The grey literature search focused on relevant websites, while the Google searches focused on identifying specific interventions by region. We searched the following sources for relevant papers: The European Association for Grey Literature Exploitation (EAGLE); The System for Information on Grey Literature in Europe (SIGLE, a bibliographic database); documents and meeting reports from the World Bank and World Health Organization (WHO) websites. The exclusion criteria that was used for the peer-reviewed literature was utilised for the focused Google searches. These focused Google searches were completed between November 2017 and March 2018 with the following search string format: “[intervention name]” costs [one of the following: Africa, Asia, East Europe] -US (where “-” is the Boolean term for minus) [5]. We reviewed the first 50 documents that resulted from the algorithm used in Google for different websites (see Supplementary Table 1 in the electronic supplementary material [ESM]). The final number of grey literature papers identified as potentially containing TB unit cost data was 31 (8%). We excluded duplicates and followed the same screening as for the peer-reviewed literature. When an abstract was not available, the executive summary was assessed.

We expected that most unit costs would be sourced from papers with noneconomic primary outcomes, as there have been very few costing studies in TB with large sample sizes. Searches were purposively broad to encompass as many papers with costs as possible whether their primary outcome was economic or noneconomic. This increased the number of papers that were not relevant and so care was taken to ensure that the exclusion criteria were correctly applied.

#### Data Extraction

We developed a comprehensive extraction tool in Microsoft Excel as part of the GHCC [5], which allowed us to describe the methods used, appraise the quality and reporting standards of included papers, and extract costs (not presented here). The extraction tool was developed by GHCC in order to assess quality and compliance with the GHCC Reference Case for Estimating the Costs of Global Health Services and Interventions [3] and was aligned with research needs (paper metadata and methodology) while also optimising data collection for open access publication in the Unit Cost Study Repository on the GHCC website (which displays fully disaggregated cost data).

Categorisation of unit costs was done according to GHCC’s intervention-standardised unit cost typology. Unit cost data were allocated to one of six TB interventions: above-service costs, active case finding (ACF), intensified case finding (ICF), passive case finding (PCF), TB prevention and TB treatment (see Supplementary Table 2 in the ESM). TB case detection and diagnosis can be done in three different ways; through passive case finding, which is the detection of tuberculosis cases where individuals self-identify at health facilities; active case finding, which is detection of cases of those not in health facilities, such as through outreach services; and intensified case finding, which is detection of cases in high-risk populations (see ESM for further explanation). Unit costs were further allocated to standard categories including geography (e.g. country, urbanicity), target population (e.g. demographic, clinical), implementation (e.g. platform, ownership, target populations, technology), costing methodology (e.g. perspective, economic or financial cost), and detailed information about how the intervention and study were conducted (e.g. year of the cost data collection, discount rate). Data were extracted on study scope, sampling, methods, inclusion of costs, valuation and analysis. We assessed whether specific information was explicitly stated, easily inferable or not reported (NR). Double extraction was performed by two teams of two extractors each, with significant interaction among the extractors. Further quality assurance work was undertaken by a senior researcher.

A study quality rating system was developed and applied to this database (and to the HIV costing papers on the GHCC Unit Cost Study Repository, which is available online) [5]. This study quality rating system assesses four categories and results in a composite quality rating made up of four letters for which there are four levels for each (A–D), with A representing the highest quartile of scores and B–D representing the three successively lower quartiles (see Table 2 for the quality rating of each paper in this review). The four quality rating indicators have equal weighting for assessing quality. AAAA would be the highest composite score and DDDD would be the lowest possible composite score based on this system. Under the first category, *Key Cost Components* (note different terms/labels were used in the original GHCC study quality rating system. ‘Bias low’ has been changed to *Key Cost Components* in this review and ‘Bias high’ has been changed to *Bias*), a paper would receive an ‘A’ if they appropriately account for and report key cost components such as above-service delivery cost, overhead costs, personnel inefficiency/downtime adjustment, and value volunteer time. This provides a signal for the completeness of the cost estimated. For the second category, *Bias*, an ‘A’ is given if the paper appropriately annuitises capital costs (which is deemed appropriate depending on the stated time since the programme started) and omits unrelated costs, for instance unrelated research costs. These are not the only sources of bias in costing papers, but as with the *Key Cost Components,* these indicators are considered a signal of potential bias. For the third category, *Precision*, an ‘A’ is indicated if the paper follows sampling, data collection and reports the cost estimate in a way that correctly reflects the level of precision of the paper. These include sampling at a country or site level as appropriate, selecting and reporting on a relevant cost allocation method, resource identification, the method of measuring output, and the number of sites selected. The fourth category is *Reporting*. An ‘A’ would be given for this category if the authors explicitly report key methods and results, such as the urbanicity (rural, urban, peri-urban or a mixture), the ownership (public, NGO, etc.), intervention components and breakdown by activity. In all the categories outlined above, completely omitting or failing to account for these aspects would result in scoring a ‘D’. Being given a low score for *Key Cost Components* could indicate an underestimation of unit costs, while a low score for *Bias* could indicate an overestimation (for instance, if the paper inappropriately included research costs, did not amortise or only costed the initial time period (< 6 months) of a new intervention or programme). The scoring rubric can be found in Supplementary Table 3 (see ESM) and further information on this quality rating can be found in the paper “Developing the Global Health Cost Consortium Unit Cost Study Repository for HIV and TB: Methodology and Lessons Learned” [5].

**Table 2:** List of all papers in review including final quality rating

Interventions	Quality: final study rating	Number of unit costs	Sites	Countries	Lead author	Reference year	Reported currency year
TB treatment	AADB	9	20	South Africa	Vassall et al. [54]	2017	2014
TB treatment	ABAA	23	8	Brazil	Trajman et al. [60]	2016	2013
TB treatment	ABDA	2	1	Nigeria	Musa et al. [61]	2016	2014
TB treatment	AADB	3	1	South Africa	Naidoo et al. [62]	2015	2009
TB treatment	AADB	2	1	South Africa	Sinanovic et al. [50]	2015	2013
TB treatment	AACC	1	5	Nigeria	Adewole et al. [63]	2015	2014
TB treatment	ABDB	14	4	China	Fitzpatrick et al. [25]	2015	2011
TB treatment	AADB	2	NR	South Africa	Cox et al. [64]	2015	2013
TB treatment	AADA	3	1	Malaysia	Atif et al. [43]	2014	2013
TB treatment	ACBA	6	1	China	Xia et al. [65]	2014	2009
TB treatment	ABDB	3	NR	China	Wang et al. [27]	2014	2008
TB treatment	ABCA	18	1	Kazakhstan	Maimakov et al. [66]	2013	2013
TB treatment	ABDB	7	1	South Africa	Pooran et al. [29]	2013	2011
TB treatment	ABCA	9	51	China	Zou et al. [14]	2013	2008
TB treatment	ABDB	5	1	South Africa	Schnippel et al. [15]	2013	2011
TB treatment	ABCA	2	7	Yemen	Othman et al. [67]	2012	2009
TB treatment	ABAA	1	1	South Africa	Janson et al. [68]	2012	2009
TB treatment	AABA	5	27	Nigeria	Umar et al. [69]	2011	2008
TB treatment	ABDB	2	2	Brazil	do Prado et al. [70]	2011	2006
TB treatment	ABDC	2	NR	Ethiopia	Datiko et al. [71]	2010	2007
TB treatment	ACDB	4	NR	India	Pantoja et al. [72]	2009	2005
TB treatment	CADA	8	NR	Indonesia	Johns et al. [73]	2009	2005
TB treatment	AAAB	4	11	Ukraine	Vassall et al. [55]	2009	2003
TB treatment	ABCA	7	3	Nepal	Mirzoev et al. [16]	2008	2002
TB treatment	ABAA	1	1	Malaysia	Elamin et al. [74]	2008	2003
TB treatment	CADB	8	1	Nepal	Karki et al. K. [75]	2007	2006
TB treatment	ABCA	6	1	South Africa	Sinanovic et al. [52]	2006	2001
TB treatment	ABDB	25	NR	Russian Federation	Atun et al. [76]	2006	2000
TB treatment	ABDB	23	NR	Sudan	El-Sony et al. [56]	2006	2005
TB treatment	AACA	5	NR	India	Floyd et al. [44]	2006	2002
TB treatment	ABBA	6	1	South Africa	Sinanovic et al. [51]	2006	2001
TB treatment	AAAA	1	1	Philippines	Tupasi et al. [58]	2006	2002
TB treatment	ABCA	9	1	Brazil	Costa et al. [77]	2005	1999
TB treatment	AABA	4	5	United Republic of Tanzania	Wandwalo et al. [18]	2005	2002
TB treatment	AACB	5	149	India	Muniyandi et al. [57]	2005	2002
TB treatment	ABCB	8	NR	South Africa	Sinanovic et al. [49]	2003	1997
TB treatment	ABCA	5	5	Zimbabwe	Hongoro et al. [78]	2003	1999
TB treatment	AABA	4	NR	Malawi	Floyd et al. [45]	2003	1998
TB treatment	AABA	2	NR	Botswana	Moalosi et al. [79]	2003	1998
TB treatment	ABDA	4	2	Kenya	Nganda et al. [80]	2003	1998
TB treatment	AABA	4	NR	Uganda	Okello et al. [19]	2003	1998
TB treatment	ABDA	2	2	Bangladesh	Islam et al. [21]	2002	1997
TB treatment	ABDA	10	1	Russian Federation	Jacobs et al. [81]	2002	1997
TB treatment	ABDB	20	4	Thailand	Kamolratanakul et al. [46]	2002	1997
TB treatment	AACB	3	3	Pakistan	Khan et al. [23]	2002	1998
TB treatment	ABAA	1	21	Peru	Suárez et al. [20]	2002	2000
TB treatment	AABC	4	1	South Africa	Dick et al. [82]	1998	1994
TB treatment	ABDB	2	1	South Africa	Floyd et al. [12]	1997	1996
TB treatment	AADA	4	1	South Africa	Wilkinson et al. [22]	1997	1994
TB treatment	ABAA	1	NR	Thailand	Sawert et al. [83]	1997	1995
TB treatment	ABCB	2	1	Uganda	Saunderson et al. [28]	1995	1992
TB treatment	ABBA	4	5	Thailand	Kamolratanakul et al. [47]	1993	1989
TB treatment	AACC	4	4	Thailand	Chunhaswasdikul et al. [84]	1992	1991
TB treatment	AACB	12	3	Malawi, Mozambique, United Republic of Tanzania	Murray et al. [10]	1991	1990
TB prevention	AABA	4	29	Brazil	Azadi et al. [35]	2014	2010
TB prevention	AABB	4	1	Zambia	Terris-Prestholt et al. [38]	2008	2007
TB prevention	ABAA	1	1	Uganda	Shrestha et al. [85]	2007	2003
TB prevention	ABDB	2	2	Uganda	Aisu et al. [36]	1995	1992
PCF	AADB	11	1	Malawi	Nliwasa et al. [30]	2016	2014
PCF	ABBA	22	21	South Africa	Cunnama et al. [24]	2016	2013
PCF	ABCA	9	5	Uganda	Hsiang et al. [86]	2016	2014
PCF	AADB	8	1	Cambodia, Georgia, Kenya, Eswatini	Page et al. [8]	2015	2014
PCF	AACC	6	5	Malawi	Zwerling et al. [87]	2015	2010
PCF	ABDB	4	1	South Africa	van Rie et al. [13]	2013	2010
PCF	ABCC	8	4	China	Pang et al. [88]	2013	2011
PCF	AABB	2	1	United Republic of Tanzania	Kidenya et al. [89]	2013	2011
PCF	AADB	3	1	Brazil	Guerra et al. [90]	2013	2012
PCF	AADC	3	1	South Africa	Dorman et al. [91]	2012	2011
PCF	AADB	2	NR	South Africa	Schnippel et al. [48]	2012	2011
PCF	AACA	2	2	South Africa	Whitelaw et al. [92]	2011	2010
PCF	ACDB	7	10	Ethiopia	Mesfin et al. [93]	2010	2005
PCF	ACDB	3	20	South Africa	Fairall et al. [94]	2010	2009
PCF	ABDB	5	1	Uganda	Ogwang et al. [31]	2009	2005
PCF	AABB	10	37	Peru	Acuna-Villaorduna et al. [32]	2008	2004
PCF	ABDA	2	1	Kenya	van Cleeff et al. [53]	2005	2004
PCF	ABDA	2	1	Kenya	van Cleeff et al. [33]	2005	2004
PCF	ABAA	1	1	Zambia	Walker et al. [95]	2000	1998
PCF	AADB	2	1	Kenya	Roos et al. [34]	1998	1997
ICF	ABAA	1	1	Botswana	Smith et al. [96]	2015	2010
ICF	ABCA	8	NR	Kenya	Yakhelef et al. [97]	2014	2009
ICF	CABB	2	3	South Africa	Peter et al. [98]	2013	2012
ICF	AADB	3	1	Thailand	Ngamlert et al. [99]	2009	2007
ICF	ABCA	4	29	Brazil	Dowdy et al. [100]	2008	2006
ICF	AADC	14	1	South Africa	Hudson et al. [101]	2000	1995
ACF	ABAA	1	NR	Cambodia	Yadav et al. [26]	2014	2012
ACF	AACC	4	5	Cambodia	Eang et al. [102]	2012	2010
ACF	ABCB	3	1	South Africa	Chihota et al. [103]	2010	2007
Above-service costs	AACB	4	5	Nigeria	Abdurrahman et al. [11]	2014	2012
ACF, TB treatment	CABB	3	4	South Africa	Zishiri et al. [104]	2014	2013
ACF, TB treatment	AADB (AADA for TB treatment component)	6	1	Tajikistan	Winetsky et al. [105]	2012	2009
ACF, TB treatment, TB prevention	AADB	4	NR	Brazil	Steffen et al. [40]	2013	2010
ACF, TB prevention	AADB	2	1	Malaysia	Atif et al. [42]	2012	2010
ICF, TB treatment	ABCC	2	1	South Africa	Kranzer et al. [106]	2012	2011
ICF, TB prevention	ABCA	6	1	South Africa	Hausler et al. [37]	2006	2002
PCF, TB treatment	ABDA	7	NR	South Africa	Meyer-Rath et al. [107]	2012	2011
PCF, TB treatment	AADA (AADB for TB treatment component)	4	11	Indonesia	Mahendradhata et al. [108]	2010	2005
PCF, TB treatment	ABAB (ABDA for TB treatment component)	2	16	Haiti	Jacquet et al. [17]	2006	2003
PCF, TB treatment	AABB	32	NR	Syrian Arab Republic, Egypt	Vassall et al. [9]	2002	1999
PCF, TB treatment	AABB	29	NR	Russian Federation	Khomenko A.G. [109]	1998	1996
PCF, TB treatment, TB prevention	ABCB	14	NR	South Africa	Mandalakas et al. [41]	2012	2009
PCF, ICF, ACF	AACA (AACB for ACF component)	3	1	Uganda	Sekandi et al. [110]	2015	2013
PCF, ACF	ABBC	8	105	South Africa	Clarke et al. [111]	2006	2004
TB prevention, PCF, TB treatment	ABDB (ABDC for PCF component)	7	NR	Botswana	Samandari et al. [39]	2011	2008

*ACF* active case finding, *ICF* intensified case finding, *NR* not reported, *PCF* passive case finding, *TB* tuberculosis

#### Data Analysis

We conducted a descriptive analysis looking at study characteristics and frequencies of reporting that met the GHCC Reference Case [3] checklist (see Supplementary Table 4 in the ESM). The 17 principles of the GHCC Reference Case [3], which cover study design, resource use measurement, application of pricing and valuation and application of reporting and analysis, were kept in mind when developing the data analysis. Many of the variables analysed were included in the study quality rating system; however, this descriptive analysis was done separately to the application of the quality rating.

In addition, to investigate the indicators of reporting quality (variables in a costing that may result in a higher reporting quality rating), we used R programming in RStudio to perform basic machine learning to determine which variables (features) were important in the reporting quality rating. Machine learning is a way to understand the structure of the data. It automates a process, in the case of this review, of predicting a categorical variable to develop a classification tree based on decision rules (an algorithm). Hence, it takes the full dataset and tries to classify it as best it can into subsets so that the data in each subset is as homogenous as possible. A classification tree starts with a node that represents all the data at that point, and then branches out with a decision being made at every branch point. It has the strength of being easy to interpret by looking at the tree. However, it has the drawback that small changes in the data can result in very different trees.

This machine learning meant taking the reporting quality score variable (either an ‘A’, ‘B’ or ‘C’ as no papers received a ‘D’) and objectively ascertaining which of a subset of variables was the most important in influencing this score in order to comment on which aspects should be focused on in future studies. This method of recursive partitioning (utilising the ‘rpart’ package) allows one to train a classification model using the data and then evaluate that model with a retained portion of that same database. Recursive partitioning means that the model takes the data and splits it based on the variables in the model in order to better understand the variable of interest, in this case the reporting quality variable.

**Fig. 1:**
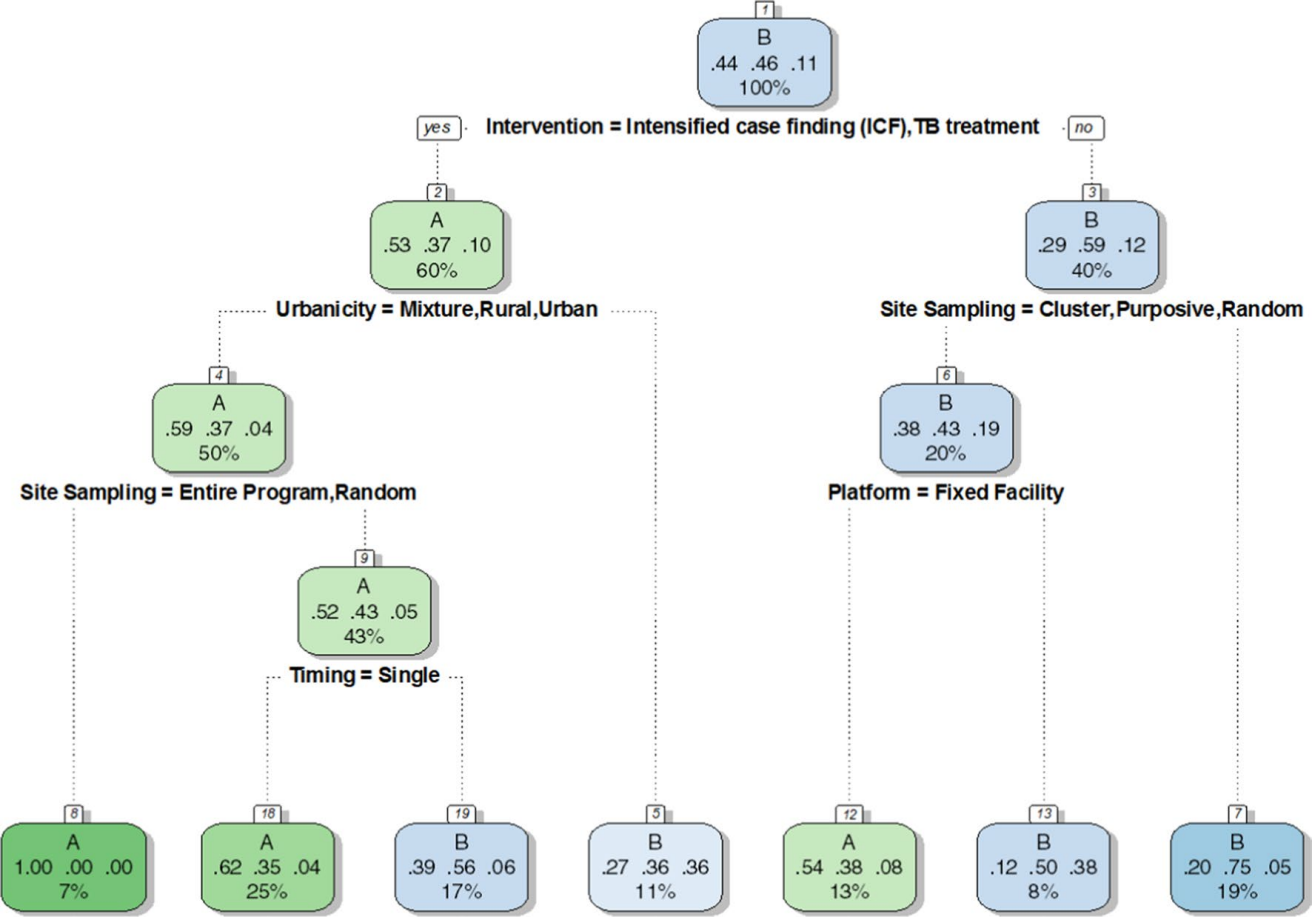
Classification tree. The label for the node reflects the dominant class—i.e. node 2 = A. The next set of numbers in the node are the proportion of the classes in the node—i.e. node 2 = 53% A, 37% B and 10% C (note the proportions here are conditional on the decisions that have already been made to reach that point in the tree). The final number in the node is the proportion of data in the node—i.e. node 2 = 60% of the data (40% falls in node 3)

The database was relatively small (only 103 rows and 140 variables/features) and within each variable there was a high level of variability. After exploratory analysis, variables were limited to a subset of study characteristics and methods used in papers (see Table 3), with complete data and less variability in their categorisation, that were thought to be important in predicting reporting quality (for instance, the disaggregated unit cost data in US dollars was not included and neither were more subjective variables such as which costs were omitted and the justification for this). The model was then trained using all the papers (103) in the database and subsequently a random 20% (of the same dataset) was utilised for testing. A confusion index was used to assess accuracy in the predictive power of the model, which takes the known values (from the training data) and predicted values from the model and tests how well the model predicts the reporting quality rating using the test data. Diagrammatically this model is visualised as a classification tree (see Fig. 1).

**Table 3:**
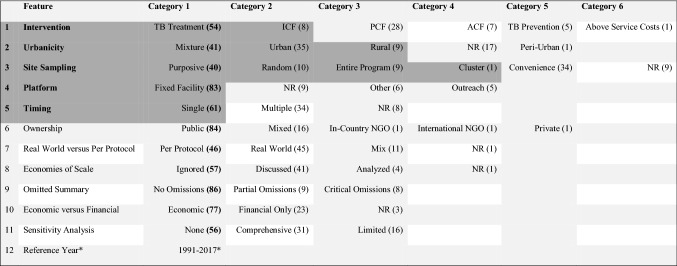
Key features with number of papers by category in brackets

The features that were more influential in the reporting quality rating (found through recursive partitioning) are highlighted in dark grey

*ACF* active case finding, *ICF* intensified case finding, *NGO* non-governmental organisation, *NR* not reported, *PCF* passive case finding

*For Reference year categories see Fig. 4, which displays all 22 options between 1990 and 2018

## Results

### Paper Selection

**Fig. 2:**
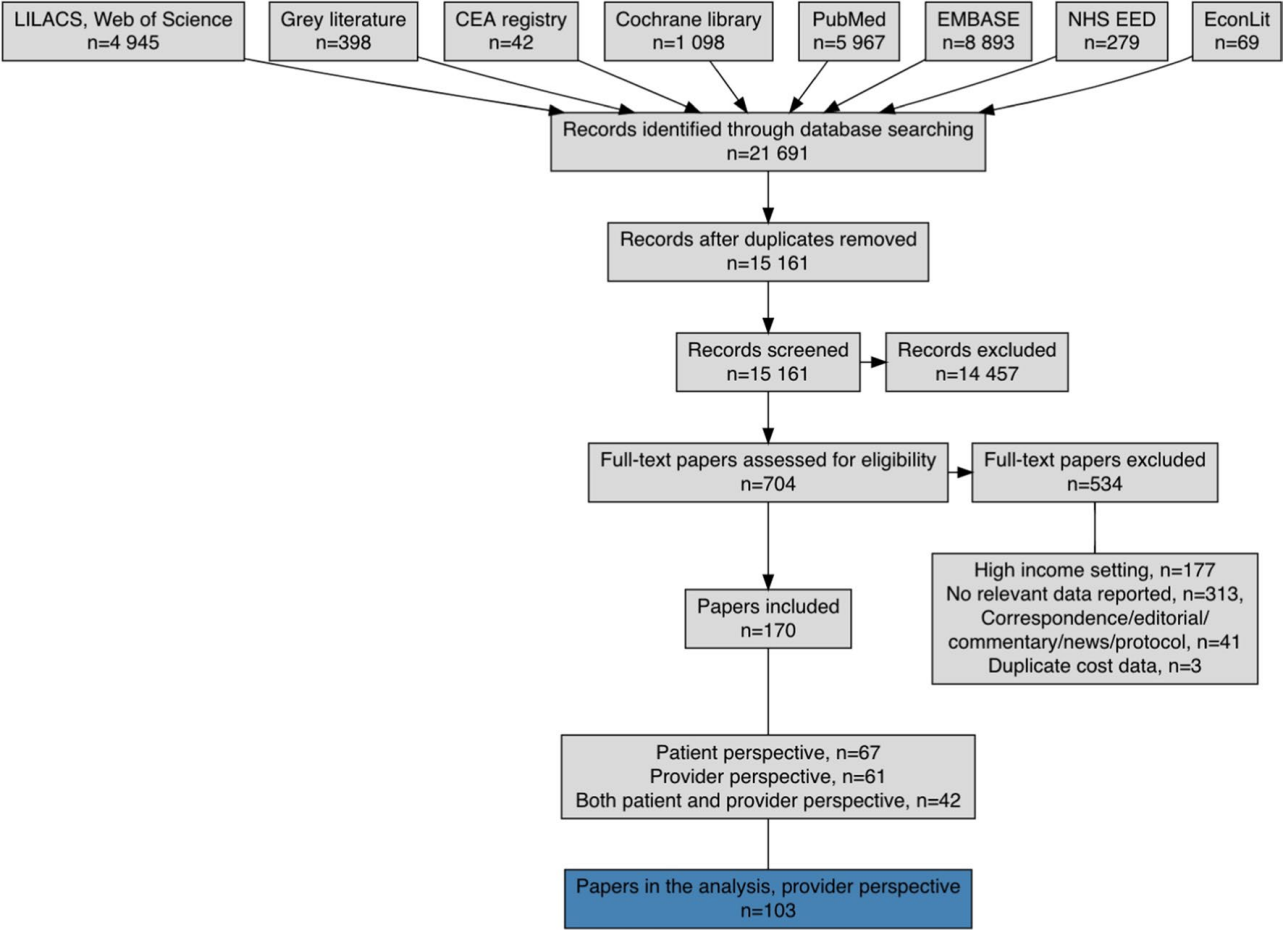
PRISMA flowchart

**Fig. 3:**
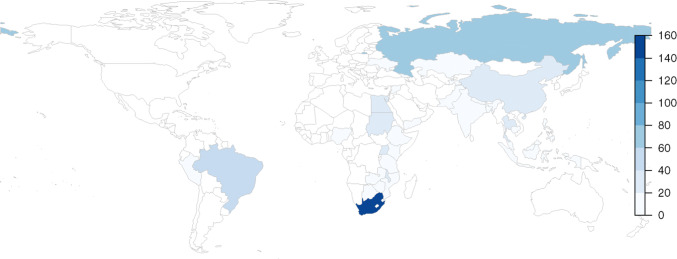
Number of unit costs for TB interventions available in low- and middle-income countries

We identified 21,691 papers through our search strategy. Overall, a total of 15,161 papers were identified after excluding duplicates (see Fig. 2 for results by database). The systematic searches described above resulted in 170 papers containing empirically collected patient and provider costs relating to TB in low- and middle-income countries. Data from 103 cost papers (See Table 2) relating to TB costs using the provider perspective were included, representing 627 unit costs for 34 countries (see Fig. 3 and Supplementary Table 5 [ESM]).

### Study Characteristics

The costing purpose of the studies was extracted for each paper and was quite diverse given that the studies were a mix of costing done at different health facility levels, in different settings, for the purposes of economic evaluation, priority setting, budgeting, financial planning and technical efficiency analysis (Supplementary Table 4, see ESM).

Table 4 shows the number of papers and the number of unit costs by type of intervention (Supplementary Table 5 indicates the number of papers and the number of unit costs for all 34 countries, see ESM). Some papers reported on several unit costs across multiple interventions and several countries. There were three multi-country studies [8–10] covering Cambodia, Georgia, Kenya and Eswatini [8]; Syrian Arab Republic and Egypt [9]; and Malawi, Mozambique and United Republic of Tanzania [10]. Only 18 of the 30 high TB burden countries (60%) (see Table 5) as listed by WHO [1] have unit costs, while TB interventions in the remaining 12 high burden countries (to our knowledge and for the search time period) have not been costed or there are no costs in the public domain (Angola, Central African Republic, Congo, Democratic People’s Republic of Korea, Democratic Republic of the Congo, Lesotho, Liberia, Myanmar, Namibia, Papua New Guinea, Sierra Leone and Vietnam). These 18 high burden countries represent 75 papers in the database (73%, i.e. 27% of papers are for countries that are not on the high TB burden list) and 443 unit costs (71%). South Africa has the highest number of TB unit costs in the dataset (154 unit costs from 28 papers, see Table 5 and Fig. 3). These cover ACF, PCF, ICF, TB treatment and TB prevention, but no papers explicitly look only at above-service costs.

**Table 4:** Representation of intervention unit cost by articles/reports

Type of interventions	Articles/reports	Unit cost estimates
Above-service costs	1	4
Active case finding (ACF)	9	20
Intensified case finding (ICF)	9	36
Passive case finding (PCF)	29	139
TB prevention	9	19
TB treatment	65	409
Total	122	627

*TB* tuberculosis

**Table 5:**
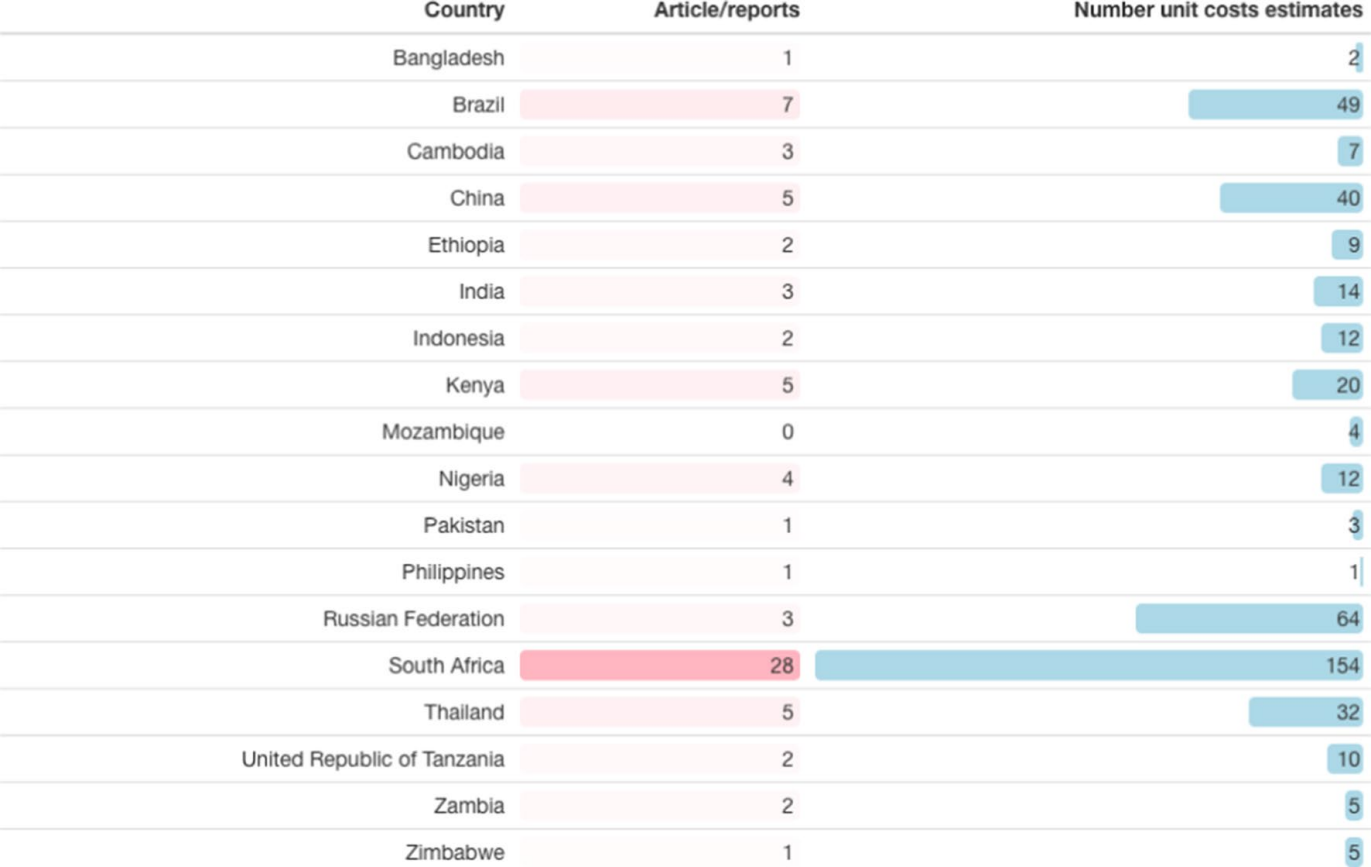
Visual representation of the number of tuberculosis papers and unit costs for countries on the World Health Organization’s 30 high burden country list

**Fig. 4:**
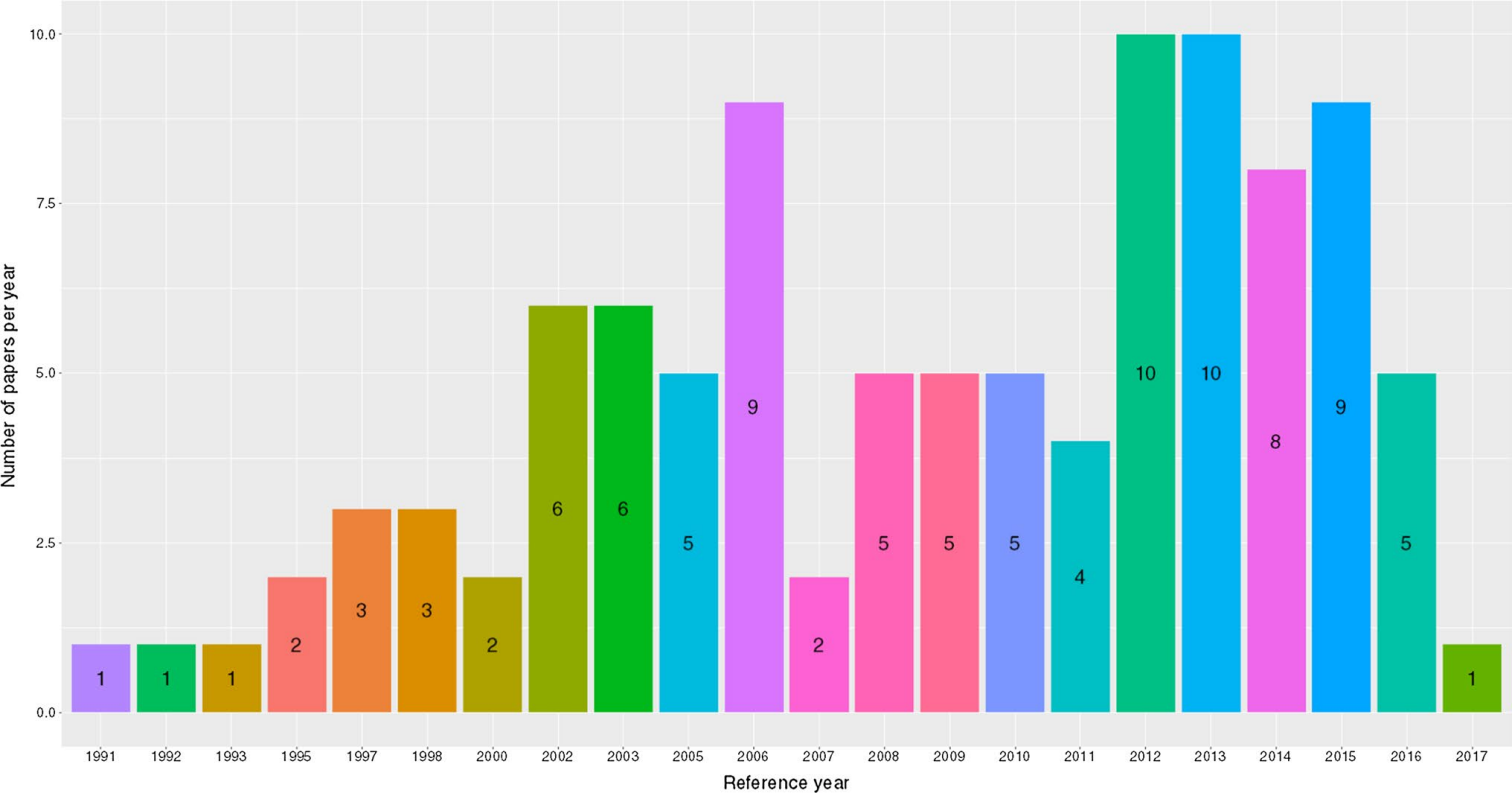
Represented years

The period between 1991 and the end of 2005 accounts for 29% of the papers obtained and extracted (Fig. 4). There were no costing papers identified for TB vaccination as an intervention, nor as a technology under TB prevention (this despite further targeted searching for Bacillus Calmette–Guérin [BCG] vaccination costing work). Only one paper estimated above-service costs [11] as its main intervention in the past decade, and none were calculated prior to this. There were 19 papers that identified and listed some above-service-level costs [12–29], but these were not the core intervention reported on. Unit costs for ACF have only been estimated from 2006 onwards and remain underrepresented compared with PCF and TB treatment. There were papers looking at the costs of ICF from as early as 2000, but again are underrepresented (compared with PCF and TB treatment). The majority of the unit costs (87% together) in this dataset are for the two interventions of TB treatment (409 unit cost estimates) and PCF (139 unit cost estimates) (see Table 4). Extracted unit costs were not distributed equally across included papers. For example, within technology types for PCF interventions, there is only one paper that looks at loop-mediated isothermal amplification (LAMP) [30], two that look at line-probe assay (LPA) [31, 32], and three that assess polymerase chain reaction (PCR) [24, 33, 34]. In comparison, among papers that included unit costs for TB prevention, 63% of the unit costs are provided by seven papers assessing isoniazid preventive therapy (IPT) [35–41] in five countries, only three of which are from the 30 high burden countries.

The mean time lag between reported currency year and publication of costs (reference year) was 3 years with a standard deviation of ± 1.6 years, a maximum of 6 and a minimum of 0 years. Seven lead authors have more than one paper in this dataset: Atif [42, 43], Floyd [12, 44, 45], Kamolratanakul [46, 47], Schnippel [15, 48], Sinanovic [49–52], van Cleeff [33, 53] and Vassall [9, 54, 55]. Of the 560 unique authors, 9% of the contributions are made by 2% of the authors (11 authors: Floyd, Sinanovic, Vassall, Churchyard, Dowdy, McCarthy, Ramma, Sanne, Schnippel, Sohn, and Stevens). There are two single-authored papers [28, 56], and one 20-author paper [25] (see Table 2).

The setting (urbanicity) for 17 papers was not reported and the predominant category (40%) used was a mixed (mixture) setting (Table 3). The majority of sampling at the site level was purposive (34 papers) or convenience (40 papers) in nature (72% together), while only 10% of papers reported random sampling methods. The main type of ownership was public (84), and fixed facility (83) platforms were the most numerous (nine papers did not comment on the platform type). Cross-sectional timing was the most frequent with 61 papers reporting this method, the remainder had multiple time points (34) or timing was not reported (8). Principle 16 of the GHCC Reference Case [3] is linked to characterising uncertainty associated with unit cost estimates; however, no sensitivity analyses were reported or undertaken for 56 papers and similarly, economies of scale were not reported in 57 papers. Papers with economic methods (77) outweighed financial (23); however, the number of papers using real-world costing was almost equal to the number of papers using a per protocol (normative) costing (45 and 46, respectively). Of those that reported the number of sites selected, the maximum number of sites in a study was 149 [57] (the minimum number was one), however the mean was 10 with standard deviation of 21 (22 papers did not report on the number of sites selected). There were 15 papers with 10 or more sites in their selection (see Table 2).

### Methods Utilised in Papers

We found there to be heterogeneity in the methods used in the papers that were reviewed. The output units were diverse even when efforts were made to group and classify these into more standard categories. Only four papers explicitly stated that research costs were included, while 10 stated that research costs were excluded (linked to Principle 6 of the GHCC Reference Case [3]). Overhead costs were included in 31 of the papers at least in part, nine reported that overhead costs were not included and 22 papers did not mention or include overhead costs. Approximately half (52 papers) had mention of the currency exchange methods, but the remainder did not. Almost a third (27% of papers) stated a discount rate, of which the majority (71% of those that reported a discount rate) cited a 3% discount rate. The division between economic and financial costing was relatively even within the database with the number of cross-sectional papers outweighing papers with multiple time points. Top-down methodology appeared to be used in nine papers, bottom-up in 27 papers, and a mixed methodology in four (use of both top-down and bottom-up elements), however, the remaining 63 papers did not state the methodology used nor could it be inferred. Cost allocations were recorded for 27 papers and were specific to study setting and context, making them difficult to standardise. Only seven papers acknowledged their amortisation methods (six were deemed to be appropriate and one stated no amortisation was undertaken), for 49 papers it appeared to be not appropriate to amortise and the remainder (47 papers) did not state their amortisation method or it could not be inferred by the reviewers. There may have been some of those that did not report their amortisation methods where amortisation was not necessary. The timing of data collection in papers was prospective in 26, retrospective in 55 and not reported (or inferable) in 22 papers (linked to Principle 10 of the GHCC Reference Case [3]). Almost two thirds (73% of papers) reported their study limitations. In terms of conflicts of interest, 42 papers (41% of the database) made a declaration, 31% of which (13 of the 42 papers) were that the authors had a conflict of interest to declare. Of the 103 papers, 61 papers did not make a declaration regarding conflicts of interest.

### Quality Rating

Only one paper received an AAAA composite quality rating score [58], while the next highest of AAAB, AABA or ABAA were received by nine papers. Nine papers were assigned an ABDC, ACDB or CADB rating and as equal weighting has been attributed to the categories these papers had the ‘lowest’ quality rating (Table 2). Overall, the highest rating for the papers in the database was given for the category of *Key Cost Components* (96% of the papers received an ‘A’), while the lowest rating was given for *Precision* (48% obtained a ‘D’ rating) (see Table 6).

**Table 6:** Quality rating as a percentage for 103 articles/reports

Indicator	A (%)	B (%)	C (%)	D (%)
Key cost components	96	0	4	0
Bias	48	49	4	0
Precision	4	20	28	48
Reporting	38	49	14	0

*Key Cost Components* (note under GHCC nomenclature is *Bias Low*), a study would receive an ‘A’ if they appropriately account for and report key cost components such as above-service delivery cost, overhead costs, personnel inefficiency/downtime adjustment and value volunteer time. This provides a signal for the completeness of the cost estimated. For the second category, *Bias* (note under GHCC nomenclature is *Bias High*), an ‘A’ is given if the study appropriately annuitises capital costs (which is deemed appropriate depending on the stated time since the programme started) and omits unrelated costs, for instance unrelated research costs. These are not the only sources of bias in costing studies, but as with the key cost components, these indicators are considered a signal of potential bias. For the third category, *Precision*, an ‘A’ is indicated if the study follows sampling, data collection, and reports the cost estimate in a way that correctly reflects the level of precision of the study. These include sampling at a country or site level as appropriate, selecting and reporting on a relevant cost allocation method, resource identification, the method of measuring output, and the number of sites selected. The fourth category is *Reporting*. An ‘A’ would be given for this category if the authors explicitly report key methods and results, such as the urbanicity (rural/urban/peri-urban or a mixture), the ownership (public, NGO etc.), intervention components and breakdown by activity. In all the categories outlined above, completely omitting or failing to account for these aspects would result in a ‘D’. Being given a low score for *Key Cost Components* could indicate an underestimation of unit costs, while a low score for *Bias* could indicate an overestimation

### Classification Model

A classification model was built as a function of 12 exogenous and endogenous variables [Reference Year, Urbanicity, Ownership, Platform, Intervention, Timing, Site Sampling, Economic versus Financial, Real World versus Per Protocol, Sensitivity Analysis, Economies of Scale, and the Omitted Summary (see Table 3)] and the resulting classification tree was constructed focusing on their link to reporting quality (see Fig. 1). It indicates the features that were more influential in the reporting quality rating (highlighted in dark grey in Table 3 and indicated in the nodes of Fig. 1); however, the accuracy (as displayed in the confusion index) when tested on 20% of the data (test data) continued to vary (from 40% to 85%) depending on the random selection of the test data from the database. Hence, the model is not a good predictor of quality, but we can take from this classification tree that these features are important signals of reporting quality rating.

The classification tree built using recursive partitioning (*n* = 103) has 13 nodes, of which seven are terminal nodes (once the data have been subset as much as is possible). The first node is a representation of all the data, with the subsequent nodes representing a categorisation or decision being made with the purpose being to subset the data repeatedly until they are as homogenous as possible. This helps us to identify which variables could be indicators of quality. The reason that the first node is blue is because there are more ‘B’s (*n* = 47; 46% of the data [written as 0.46 in Fig. 1]) than ‘A’s (*n* = 45; 44% of the data [written as 0.44 in Fig. 1]) and ‘C’s (*n* = 11; 11% of the data [written as 0.11 in Fig. 1]) in the dataset (i.e. ‘B’ is the dominant class). The probabilities in each node are conditional on the decisions that are made in the tree on the way to each node.

The features of ‘Intervention’ followed by ‘Urbanicity’ and ‘Site Sampling’ are the most likely indicators of quality of reporting, as displayed by the classification tree built using machine learning (Fig. 1). ‘Intervention’ type appears to be the most important variable for signalling a reporting quality rating (using the study quality rating system [5]), specifically if ICF or TB treatment are the interventions costed (the first branch of the tree results in 60% of the data falling in node two on the right and 40% in node three on the left. There is a 0.53 probability of receiving an ‘A’ rating for reporting as indicated in node two). If one then follows the branch to the left, the next most important aspect for obtaining an ‘A’ is ‘Urbanicity’ if rural, urban or a mixture of settings were selected as opposed to not reporting (NR) the setting or if peri-urban settings were listed; if the entire programme was assessed or random sampling was undertaken; or if a cluster, convenience, NR or purposive site sampling was used, but timing was at a single point in time, an ‘A’ rating was more likely. However, following the righthand branch, if the ‘Intervention’ was PCF, ACF, above-service costs or TB prevention, ‘Site Sampling’ was cluster, purposive or random and the ‘Platform’ was a fixed facility, an ‘A’ rating was also more likely.

## Discussion

The contribution that this review has made is exposing the availability of 103 TB unit cost papers, with 627 unit costs, where authors have taken a provider perspective for low- and middle-income countries, which have been systematically extracted from a diverse source of papers and presented for analysis using an intervention-standardised typology. The use of the study quality rating system as well as the descriptive analysis of methodology has highlighted variability in reporting, particularly around uncertainty, sampling methods and the use of top-down, bottom-up and mixed methods for cost collection. Machine learning, which to our knowledge has not previously been used to examine signals of quality in a systematic review, was utilised to identify indicators for confidence in unit costs produced.

In these data, we have identified three main gaps. Firstly, TB cost data from a provider’s perspective spans almost three decades, leaving some areas out of date; second, we have identified that 12 of the 30 high TB burden countries do not have unit costs available or these have not been reported on; and third, some TB interventions are not well represented, for instance, unit costs for TB vaccination (0% of the unit costs in this database), comprehensive above-service (level) costs, ACF, ICF and TB prevention are limited to just a handful of papers (13% of unit costs in this review).

While the purpose of the costing studies was well defined for the 103 extracted provider perspective papers, there was heterogeneity in the methods used to estimate costs, especially with respect to the methods used to measure and allocate costs. Some areas of the papers had poor reporting, such as whether ‘above facility costs’ and unrelated costs were included, which resulted in these fields having many NR (not reported) entries. Principle 13 of the GHCC Reference Case [3] assesses whether currency conversion and discount rates are clearly stated (amongst other criteria). There was under reporting of discount rates and currency conversion; however, in part this may have been that (especially with regard to discounting) it was not relevant to these studies.

In looking at the study characteristics, descriptive findings and the methodological principles as laid out by the GHCC Reference Case [3], this review of TB costing papers indicates that transparency in methods is limited due to a lack of standard reporting of methods and results. In cases where methods are reported well, there is still a variation in approaches for measuring costs observed. Variability makes comparison difficult and creates concern about which unit costs to rely on [59]. It is not just variability in methods and approaches that makes it difficult to know which costs are reliable, some costs are incomplete, others are poorly described or may have been incorrectly categorised by the authors. The findings from this review of cost methods and reporting have been used to iteratively guide the development of the GHCC Reference Case for Estimating the Costs of Global Health Services and Interventions [3] and Costing Guidelines for Tuberculosis Interventions [4]. However, as they were developed in parallel there was cross learning between the two. The GHCC Reference Case [3] encourages researchers to be explicit and open in how they estimate the cost of interventions while the Costing Guidelines for Tuberculosis Interventions inform empirical cost collection methods [4] and, subsequent to this review, two intervention categories will be added, vaccination and TB infection control and above-service-level costs may be renamed TB policy, planning, coordination and management.

Currently, the *Key Cost Components*, *Bias, Precision,* and *Reporting* indicators have equal weight, so we are unable to comment on whether one indicator is more important or relevant than another. The four dimensions of quality used in this review are quite different. Lack of completeness and bias are important issues and could indicate overall poor quality. Reporting is something that allows one to assess the quality and completeness and so is critical and hence a central focus of this review. Precision relates to sampling which has not been a historic focus of TB costing, particularly because it is common for only a few sites to be selected, often purposively or due to convenience. There were very few papers with a large number of sites and around 20% did not report on the number of sites at all; these factors are likely to have impacted on the precision. Going forward, sampling should be encouraged as well as larger TB costing studies for TB interventions where there is an identified lack of data (vaccination, above-service costs, TB prevention, ACF and ICF). It is important to keep in mind that the study quality rating system assesses the appropriateness of a paper’s methods rather than giving a definitive measure of quality or causation.

Understanding the quality of cost data and reporting can help to inform which methods need strengthening and what areas of reporting should be standardised. When we used machine learning and ran the model repeatedly, the accuracy level, as determined by using a confusion index, fluctuated markedly indicating that the model did not reliably predict that a paper would score a particular rating (‘A’, ‘B’ or ‘C’, no papers received a ‘D’ for this category) for reporting due to the variability within the features and due to limited training given the comparatively small dataset. The machine learning was focused on the reporting of these variables. However, from this analysis, papers that report on ‘Intervention’ (in particular), ‘Urbanicity’, ‘Site Sampling’, ‘Platform’ and ‘Timing’ were identified by the machine learning model to be more indicative of the quality of reporting standards overall in comparison to reporting on ‘Ownership’, Real World versus Per Protocol Costing’, ‘Economies of Scale’, ‘Omissions’, ‘Economic versus Financial’ costing, ‘Sensitivity Analysis’ and the ‘Reference Year’.

The economic implications of this review are that TB unit costs have been identified, aligned to intervention-standardised typology and placed in an open access Unit Cost Study Repository. These easily accessible data are particularly useful for modelers and TB decision makers. The indication from machine learning is that reporting on ‘Intervention’ (in particular), ‘Urbanicity’ and ‘Site Sampling’, can guide those undertaking costing work, which often leads into National Strategic Planning, economic evaluation, budgeting and priority setting.

There are of course a couple of considerations, namely that the database primarily assesses what has been reported, so something may have been done and not written down or may have been reported but may have only been done at a superficial level. The other consideration is that the reporting quality rating is determined by the data that are included and then some of those aspects are assessed again in the recursive partitioning, which could have led to overfitting of the model. The database did require inference by the study team when authors had not been explicit in their reporting and so there could also have been limitations in the fields used to assess quality or even in the choice of fields to include in the quality rating. The search strategy may have been a limiting factor in the uncovering of TB vaccination costs (BCG in particular); however, a separate targeted search was conducted specifically for this and no empirical TB costs were found. Since the completion of the search a valuable database of vaccination costs, called the Immunization Delivery Cost Catalogue, has been made publicly available and can be found online at: http://immunizationeconomics.org/ican-idcc. This catalogue illuminated that the search for BCG costs would need to be aimed at childhood vaccinations, which often form part of an immunisation programme or package of vaccines, and so we were unlikely to pick up studies related to BCG with our selected search terms. In future reviews, we suggest using more search terms linked to TB vaccination and TB infection control, which have been added to the GHCC intervention-standardised list subsequent to this review.

## Conclusion

This study demonstrates the variability in reporting and methods used to estimate TB costs from a provider perspective. Those working in TB and TB decision making would benefit if researchers explicitly reported what they did and increased their transparency regarding the methods they used to estimate the costs of TB interventions. We suggest being unambiguous in the methods used such as reporting mixed or separate top-down and bottom-up costing, and standardising unit costs to ease comparison between different papers’ findings (Supplementary Table 4, see ESM). A set of guidelines for TB costing drawing from this review further explains the important methodological aspects one should focus on [4]. Going forward, the unit cost gap needs to be addressed for TB vaccination (especially new vaccine candidates as they emerge), above-service costs, ACF, ICF and TB prevention and the remaining 12 high burden countries without TB unit costs in literature (Angola, Democratic People’s Republic of Korea, Democratic Republic of the Congo, Myanmar, Vietnam, Central African Republic, Congo, Lesotho, Liberia, Namibia, Papua New Guinea and Sierra Leone).

## Electronic supplementary material

Below is the link to the electronic supplementary material.

Supplementary material 1 (DOCX 4289 kb)

## Data Availability

An open access version of the database can be found at in the Unit Cost Study Repository https://ghcosting.org/pages/data/ucsr/app/. Please contact the corresponding author for R Scripts relating to the analysis.
