# Correction to: A Systematic Review of Methodological Variation in Healthcare Provider Perspective Tuberculosis Costing Papers Conducted in Low- and Middle-Income Settings, Using An Intervention-Standardised Unit Cost Typology

**DOI:** 10.1007/s40273-020-00928-0

**Published:** 2020-05-27

**Authors:** Lucy Cunnama, Gabriela B. Gomez, Mariana Siapka, Ben Herzel, Jeremy Hill, Angela Kairu, Carol Levin, Dickson Okello, Willyanne DeCormier Plosky, Inés Garcia Baena, Sedona Sweeney, Anna Vassall, Edina Sinanovic

**Affiliations:** 1 Health Economics Unit, School of Public Health and Family Medicine, Faculty of Health Sciences, University of Cape Town, Anzio Road, Cape Town, South Africa; 2 Department of Global Health and Development, Faculty of Public Health and Policy, London School of Hygiene and Tropical Medicine, London, UK; 3 Institute for Health Policy Studies, University of California, San Francisco, CA USA; 4 Department of Global Health, University of Washington, Seattle, WA USA; 5 Avenir Health, Glastonbury, CT USA; 6 TB Monitoring and Evaluation (TME), Global TB Programme, The World Health Organization, Geneva, Switzerland

## Correction to: PharmacoEconomics (2020) 10.1007/s40273-020-00910-w

In the original version of this article, Fig. 3 was published in an incorrect format. The correct figure is published with this correction.

**Fig. 3:**
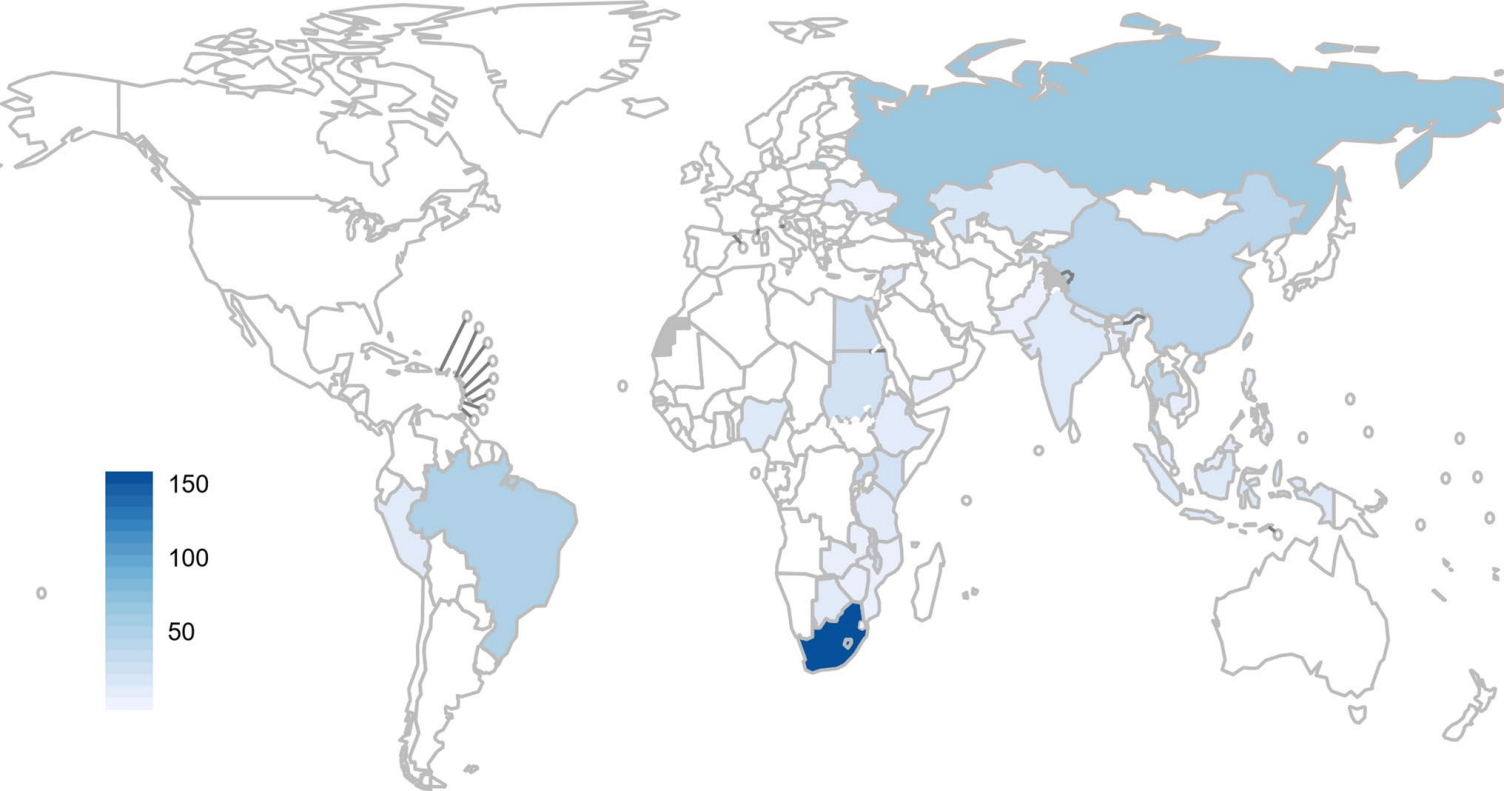
Number of unit costs for TB interventions available in low- and middle-income countries

