# Supplementary material for: A Systematic Review of Methodological Variation in Healthcare Provider Perspective Tuberculosis Costing Papers Conducted in Low- and Middle-Income Settings, Using An Intervention-Standardised Unit Cost Typology
Source: Pharmacoeconomics. 2020 May 4;38(8):819–37. doi: 10.1007/s40273-020-00910-w (PMC7437656; doi:10.1007/s40273-020-00910-w)
Supplement: Supplementary file 1 — Supplementary material 1 (DOCX 4289 kb) [file 40273_2020_910_MOESM1_ESM.docx]

# Supplementary tables:

Supplementary Table 2: Intervention-standardised TB unit costs (directly from the Costing Guidelines for Tuberculosis Interventions)(1)

Supplementary Table 3: Study quality rating system (directly (with adapted indicator labels) from Developing the Global Health Cost Consortium Unit Cost Study Repository for HIV and TB: Methodology and Lessons Learned)(2)
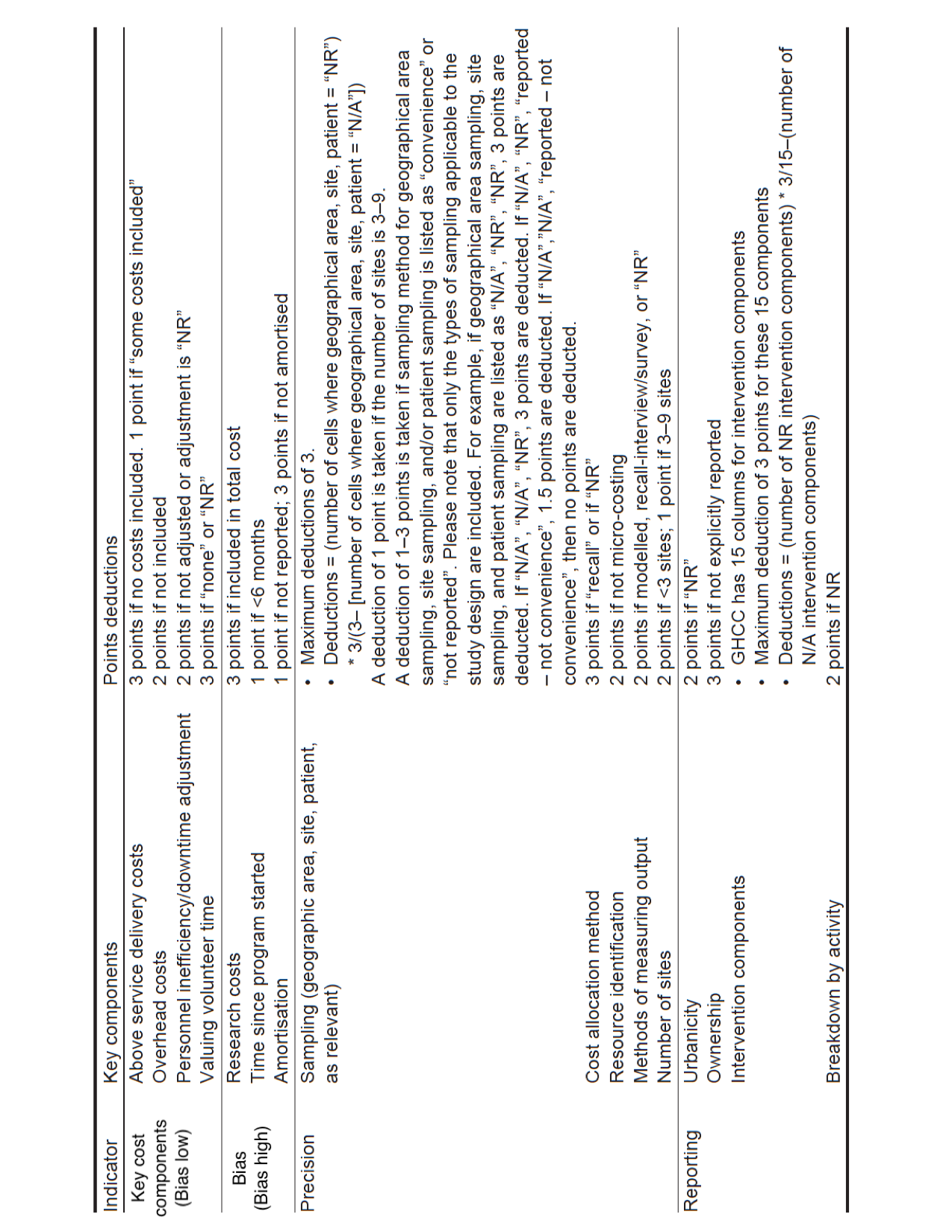


Supplementary Table 4: Principles and methods reporting checklist (directly from the Costing Guidelines for Tuberculosis Interventions)(1)

Supplementary Table 5: Number of TB papers and unit costs broken down by country
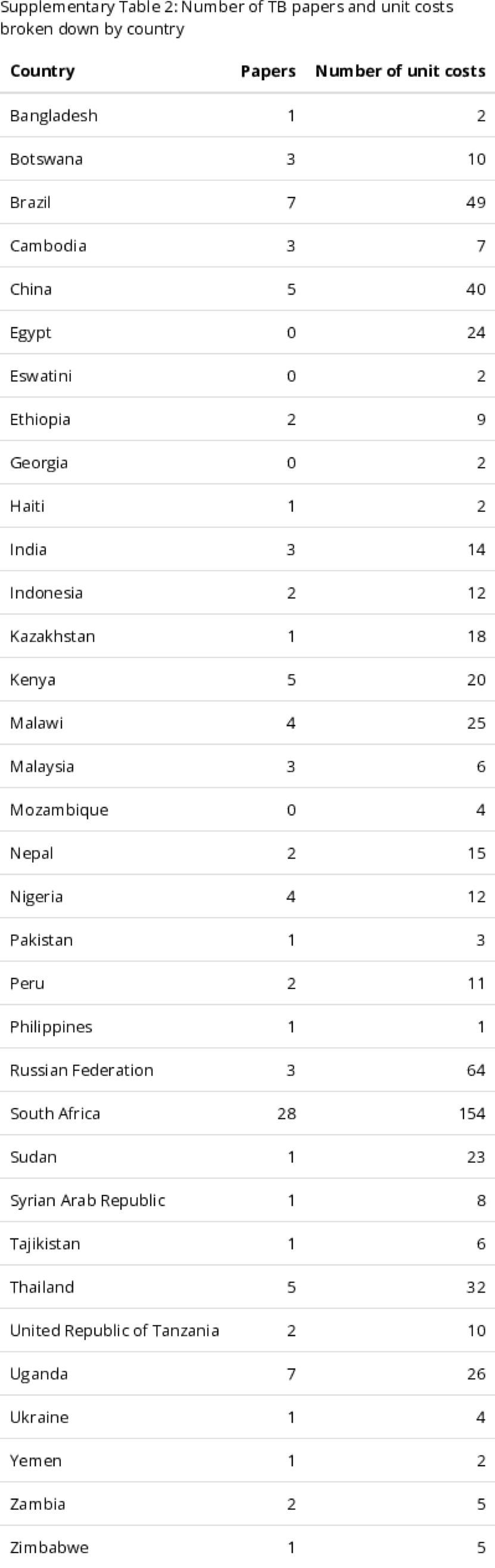


| Supplementary Table 6: how the search terms were combined with the inclusion of Medical Subject Headings (MeSH) |
| --- |
| Search terms:  cost* or economic or finance AND TB or tuberculosis or MDR#TB or XDR#TB or multi?drug or "resistant tuberculosis" or "strain resistance" or "mycobacterium tuberculosis" AND treatment or management or drugs or medication or DOTS or "directly observed treatment" or "health system*" or "hospital care" or “epidemiology" or "government hospital setting" or "community based care" or “patient* perspective" or "isoniazid preventive therapy" or "IPT" or "prevention" |
| Search details:  (cost[All Fields] OR costa[All Fields] OR costaceae[All Fields] OR costaclavin[All Fields] OR costaclavine[All Fields] OR costada[All Fields] OR costae[All Fields] OR costaii[All Fields] OR costal[All Fields] OR costal2[All Fields] OR costalgia[All Fields] OR costalis[All Fields] OR costamere[All Fields] OR costameres[All Fields] OR costameric[All Fields] OR costantinii[All Fields] OR costaricanus[All Fields] OR costaricensis[All Fields] OR costasis[All Fields] OR costata[All Fields] OR costatolide[All Fields] OR costeff[All Fields] OR costello[All Fields] OR costelytrae[All Fields] OR costen[All Fields] OR costen's[All Fields] OR costens[All Fields] OR costertonia[All Fields] OR costi[All Fields] OR costic[All Fields] OR costicola[All Fields] OR costimulates[All Fields] OR costimulation[All Fields] OR costimulator[All Fields] OR costimulatory[All Fields] OR costitutes[All Fields] OR costly[All Fields] OR costochondral[All Fields] OR costoclavicular[All Fields] OR costocoracoid[All Fields] OR costovertebral[All Fields] OR costs[All Fields] OR costsus[All Fields] OR costunolide[All Fields] OR costus[All Fields] OR costuslactone[All Fields]) OR ("economics"[MeSH Terms] OR economic[Text Word]) OR finance[All Fields] AND TB[All Fields] OR ("tuberculosis"[MeSH Terms] OR tuberculosis[Text Word]) OR "tuberculosis, multidrug-resistant"[MeSH Terms] OR ("extensively drug-resistant tuberculosis"[MeSH Terms] OR XDR#TB[Text Word]) OR multi?drug[All Fields] OR "resistant tuberculosis"[All Fields] OR (("sprains and strains"[MeSH Terms] OR strain[Text Word]) AND resistance[All Fields]) OR "mycobacterium tuberculosis"[All Fields] AND ("therapy"[Subheading] OR "therapeutics"[MeSH Terms] OR treatment[Text Word]) OR ("organization and administration"[MeSH Terms] OR "disease management"[MeSH Terms] OR management[Text Word]) OR ("pharmaceutical preparations"[MeSH Terms] OR drugs[Text Word]) OR ("pharmaceutical preparations"[MeSH Terms] OR medication[Text Word]) OR DOTS[All Fields] OR (directly[All Fields] AND observed[All Fields] AND ("therapy"[Subheading] OR "therapeutics"[MeSH Terms] OR treatment[Text Word])) OR "health system*"[All Fields] OR "hospital care"[All Fields] OR "epidemiology"[All Fields] OR (("government"[MeSH Terms] OR government[Text Word]) AND ("hospitals"[MeSH Terms] OR hospital[Text Word]) AND setting[All Fields]) OR (("residence characteristics"[MeSH Terms] OR community[Text Word]) AND based[All Fields] AND care[All Fields]) OR (("patients"[MeSH Terms] OR patient[Text Word]) AND perspective[All Fields]) OR (("isoniazid"[MeSH Terms] OR isoniazid[Text Word]) AND ("prevention and control"[Subheading] OR preventive therapy[Text Word])) OR "IPT"[All Fields] OR "prevention"[All Fields] |

Supplementary Information: Explanation of tuberculosis interventions (directly from the Costing Guidelines for Tuberculosis Interventions)(1)

**TB case detection and diagnosis: Passive case finding**

PCF is defined as detection of TB cases (screening and diagnosing of active and latent TB) reporting to public or private TB services in health facilities. The standard method of identifying people with TB is PCF, where individuals with TB symptoms present themselves at a health facility. A health worker assesses the person and orders a diagnostic test. Several technologies are available for PCF depending on the setting and patient attributes, including a verbal symptom screen, cough triage, sputum induction, Xpert® MTB/RIF, (light-emitting diode (LED) or Ziehl-Neelsen (ZN)) microscopy, culture (solid or liquid), film and digital x-ray, rapid HIV test, line probe assay for first (LPA-FLD) and second-line drugs (LPA-SLD), drug sensitivity testing (DST), LAMP, lateral flow urine lipoarabinomannan assay (LF-LAM), interferon-gamma release assay (IGRA) (where used for detection of latent TB infection (LTBI)), tuberculin skin test (TST, also known as Mantoux test or purified protein derivative (PPD) skin test), fine needle biopsy, bronchial and gastric lavage, and tests for extra-pulmonary TB (EPTB) (aspirates, computed tomography (CT) scan and ultrasound). Other tests include: HIV confirmatory test, cluster of differentiation 4 (CD4) count, erythrocyte sedimentation rate, total white blood cell count, full heamogram, aspartate aminotransferase (AST also known as serum glutamic-oxaloacetic transaminase (SGOT)), creatinine, creatinine clearance, glucose random blood sugar (RBS), lactic acid, lipase, thyroid stimulating hormone, body fluid analysis polymerase chain reaction – deoxyribonucleic acid (PCR-DNA) and electrocardiography (ECG). The population of concern includes both children and adults, irrespective of HIV status or TB strain. Costing should be conducted for the entire duration of the diagnostic visit, diagnostic test and patient support activity. PCF occurs in both public and private health facilities, but rarely occurs outside of the health facility or laboratory.

**TB case detection and diagnosis: Intensified case finding**

ICF detects potential active and latent TB cases among people living with HIV or diabetes or attending maternal and child health clinics or in other high-risk populations, receiving non-TB health care. Symptom screening, film or digital x-ray and Xpert® MTB/RIF are the main ICF technologies, but technologies used in PCF may also become part of the diagnostic algorithm for HIV positive adults, persons within high-risk groups (including people exposed to drug-resistant TB) attending health facilities, and children. Screening and diagnostic visits, diagnostic tests and patient support activities should be costed to obtain the unit cost of ICF.

**TB case detection and diagnosis: Active case finding**

ACF is defined as screening and diagnosing active and latent TB in those who are not in public health care (i.e. who are not detected passively). Generally, fewer technologies are used to detect cases actively, which include a verbal symptom screen, Xpert® MTB/RIF, microscopy (LED), film or digital X-ray, rapid HIV test, contract tracing, fine needle biopsy aspirates, CT scan or ultrasound. Other tests can include culture (solid or liquid), LPA, DST, IGRA, TST, and bronchial or gastric lavage. It can be conducted in private or public facilities, in mobile clinics, through outreach programmes such as to schools, prisons, or within the household. Target populations are household contacts (adults and children less than five and between five and eighteen years old), poor urban populations, prisoners, mobile populations, migrant populations, healthcare and other workers with an occupational risk of contracting TB. Any new ACF technologies should be costed during the start-up as well as the continued implementation for a patient or an episode. The activities included in ACF are the screening visits, diagnostic visits, diagnostic tests and all patient support services.

**TB treatment**

TB treatment includes all activities involved in treating patients with active TB, observation of treatment, patient support, restoring quality of life and productivity, preventing relapse or death, reducing transmission and preventing development and transmission of drug resistance. During the intensive and continuation phases of treatment, persons of all ages being treated for either DS-TB or DR-TB (monoresistant, poly-drug resistant, Rifampicin resistant, MDR-TB, pre-XDR-TB or XDR-TB) can be treated within the household, the community, through outreach programmes, in public or private facilities, or at general or specialized TB hospitals. HIV-positive or negative patients with either pulmonary TB (PTB) or EPTB are included. In addition to the drugs for first-, second- and third-line treatment; retreatment, palliative care, monitoring tests for treatment response, adverse events, nutritional assessment, lost to follow-up tracing, ART regiment if HIV-positive and M-health are included in TB treatment.

**TB prevention**

TB prevention refers to screening and treatment to prevent latent and active TB (PTB and EPTB) amongst children and HIV-positive adults. Screening visits, treatment monitoring (including for breakthrough disease, adverse events, acquired drug resistance), visits occur in public or private facilities. The technologies for prevention of TB include symptom screening to rule out active TB, IGRA test or TST, as well as treatment protocols of isoniazid for six months (6H), lifelong isoniazid (H), three months of once-weekly isoniazid and rifapentine (3HP), or rifapentine only, ART regimen for HIV positive patients and cotrimoxazole prophylaxis.

**TB programme above site services: Above service costs (TB policy, planning, coordination and management)**

At the national level activities and technologies that contribute to the successful delivery of TB services include development of strategic plans, TB care guidance development or adaptation, programme reviews, national and regional meetings, surveys management and information systems, supervision, procurement and supply chain management, transportation of specimens, advocacy, technical assistance, training, accreditation and quality assurance of labs, community media or information, education and communication (IEC) campaigns or any partnership activities. The delivery platforms for these activities and technologies are ministries of health, NTPs, reference laboratories, government and non-government research institutes, public health facilities, private health facilities and laboratories, regulatory bodies for food, drugs and health, NGOs, and bi- and multi-lateral partners, including WHO. Populations involved in these activities include health care workers, laboratory staff and management involved in TB or any support services.

For future categorization:

**Vaccination**

Vaccination for TB is used to prevent TB and currently includes the BCG vaccine for infants, children and young adults (between eight and eighteen years old). Treatment for adverse reactions has been included as part of the vaccination intervention.

**TB infection control**

Infection control for TB in environments such as health facilities, laboratories, congregate settings and households involves a combination of activities to minimize the risk of transmitting TB within these settings. Successful TB infection control includes early and fast diagnosis of TB, coupled with appropriate management of people with TB. Within health facilities, in addition to the administrative controls to reduce diagnostic delays and prompt treatment initiation, infection control for patients, laboratory staff and health care workers includes the use of ventilation systems, laboratory biosafety systems, UV fixtures and personal protective equipment. Congregate settings include hospital premises, prisons, refugee camps and schools. TB infection control in these settings must be coordinated with other sectors. Within households, TB infection prevention includes campaigns educating on behaviour and social change to minimize exposure. The delivery platforms include private and public health facilities, as well as facilities managed by international and national NGOs. Infection control can also occur in community-based activities and during outreach activities.

References:

1. Cunnama L, Garcia Baena I, Gomez G, Laurence Y, Levin C, Siapka M, et al. Costing guidelines for tuberculosis interventions. 2019. Licence: CC BY-NC-SA 3.0 IGO.

2. DeCormier Plosky W, Bollinger L, Alexander L, Cameron D, Carroll LN, Gomez GB, et al. Developing the Global Health Cost Consortium Unit Cost Study Repository for HIV and TB: Methodology and Lessons Learned. African Journal of AIDS Research. 2019;18(4):263-76.
